# New Appliances and Things Medical

**Published:** 1898-02-05

**Authors:** 


### NEW APPLIANCES AND THINGS MEDICAL.

**[We shall be glad to receive, at our Office, 28 & 29, Southampton Street, Strand, London, W.C., from the manufacturers, specimens of all new preparations and appliances which may be brought out from time to time.]**

### "D.C.L." MALT EXTRACT.

(The Distillers Co., Limited, Edinburgh.)

In our issue of January 23rd of last year we gave a careful analysis and report of the above malt extract. We have recently received another sample, and find that the manufacturers have in no way fallen away from the high standard of excellence which characterised their initial efforts. The extract is peculiarly agreeable to the taste, and may in fact be regarded almost more in the light of a relish or condiment than a food with medicinal properties.

### EUGOL.

(Bayard, Sons, and Bayard, 26, Bridle Lane, Golden Square, W.)

Eugol is claimed to be a scientifically prepared combination of active but non-poisonous vegetable and mineral antiseptics which can be used both internally and externally, in accordance with the directions given. It is an almost colourless and neutral liquid, having a very pleasant aromatic odour. The results of further examination show that it is, as stated, a combination of non-poisonous antiseptics. Eugol is quite miscible with water, and would be found useful in many cases where a non-poisonous and active antiseptic is essential.

### HOOBA DIGESTIVE TEA.

(Hooba Tea Co., Dunster House, Mincing Lane.)

A sample of the above tea has been forwarded us, and to satisfy ourselves of its purity and quality we have made a careful examination of its component leaves and thoroughly tested its properties as a tea suitable for invalids. In all respects we find that it comes well up to standard, possessing a delicate and pleasant aroma and being free from the astringent properties which render the coarser teas unfit for the delicate mucous membrane of the digestive system. It appears to be carefully and uniformly blended.

### COKAY.

(Allen and Hanburys, Limited, Plough Court, Lombard Street, E.C.)

Cokay is a coca wine which is said to be prepared not by the addition of cocaine or extract of coca leaves to ordinary wine, but by maceration of the finest coca leaves in an invalid wine closely resembling the well-known Imperial Tokay. Cokay is a pale-coloured wine having a very pleasant flavour in which the characteristic taste of the coca leaf is distinctly perceptible. It contained 1·0 grain of cocaine per pint, extractives 20 per cent., and alcohol equal to 31·0 per cent. of proof spirit. In every respect Cokay can be recommended as a very palatable alcoholic preparation of coca which would be preferred by many to the ordinary kinds of coca wine.

